# Preference-Based Quality of Life in Adolescents with Psychosis: Acceptability and Construct Validity of EQ-5D-3L and CHU-9D

**DOI:** 10.1192/j.eurpsy.2026.10200

**Published:** 2026-07-31

**Authors:** T. L. Andersen, N. Larsen, C. F. Nielsen, M. S. Madsen, N. K. Andersen, L. H. Hastrup, A. K. Pagsberg

**Affiliations:** 1 Child and Adolescent Mental Health Center, Copenhagen University Hospital – Mental Health Services CPH; 2 Department of Clinical Medicine, University of Copenhagen; 3 Section for Biostatistics and Evidence-Based Research, the Parker Institute, Bispebjerg and Frederiksberg Hospital, Copenhagen; 4 Psychiatric Research Unit, Psychiatry in Region Zealand, Slagelse; 5 Danish Centre for Health Economics (DaCHE), University of Southern Denmark, Odense, Denmark

## Abstract

**Introduction:**

There have only been few health economic evaluations worldwide in child and adolescent psychiatry, though they provide valuable information for optimizing services. With child and adolescent psychiatry growing as a field, there is likewise an increasing need to evaluate interventions using cost-utility analyses. For these analyses, we need appropriate preference-based quality-of-life instruments.

**Objectives:**

We evaluate the acceptability and construct validity of EQ-5D-3L and CHU-9D when self-reported by adolescents with psychosis.

**Methods:**

The sample is composed from baseline data in the OPUS YOUNG trial including 290 adolescents aged 12-17 years with first-onset psychosis. For this study, the 277 adolescents, who answered both EQ-5D-3L and CHU-9D, are included.

Danish adult tariffs are used to calculate EQ-5D-3L utility values, while Australian adolescent preference weights are used for CHU-9D. Additional measures are KIDSCREEN-10, Personal and Social Performance Scale (PSP), Clinical Global Impressions – Severity (CGI-S), and Full Scale IQ (FSIQ).

Acceptability is considered using descriptive statistics and distributions for domains and utility values. Construct validity is analyzed using Spearman correlations: Convergent validity is analyzed using KIDSCREEN-10; known-group validity is analyzed using PSP and CGI-S; and discriminant validity is analyzed using FSIQ.

**Results:**

The adolescents rated themselves better in domains phrased like mobility and self-care than domains about activities. While they rated themselves worse on sadness/depression, worry/anxiety, and tiredness, they rarely expressed lots of pain and anger. Distributions for CHU-9D had greater spread and lower ceiling effects compared to EQ-5D-3L.

The EQ-5D-3L resulted in 72 distinct utility values from -0.399 to 1 with a mean of 0.611 (SD = 0.249), a median of 0.693, nine cases with a utility less than zero and 17 cases with a utility of 1. The CHU-9D resulted in 270 distinct utility values from -0.071 to 1 with a mean of 0.423 (SD = 0.212), a median of 0.399, one case with a utility value less than zero and one case with a utility of 1.

Moderate correlations were found between EQ-5D-3L, CHU-9D, and KIDSCREEN-10 (r = 0.62 – 0.69, *p* < .001). Only weak correlations were found with PSP (r = 0.25 – 0.30, *p* < .001) and CGI-S (r = 0.21 – 0.29, p < .001). There was no correlation between EQ-5D and FSIQ (r = -0.05, *p* = .44) but a weak correlation between CHU-9D and FSIQ (r = -0.18, *p* < .01).

**Conclusions:**

EQ-5D-3L and CHU-9D can both be used for self-report by adolescents with psychosis. To optimize variance and minimize ceiling effects, levels and tariffs should be considered. Correlations show good convergent validity between health-related quality of life instruments. Though symptoms and functioning are expected to correlate with utility values, weak correlations suggests that utility should be measured separately.

**Disclosure of Interest:**

None Declared

